# Pulpless Teeth Again—Repeated Dressings

**Published:** 1897-06

**Authors:** J. W. Wassall


ARTICLE III.

### PULPLESS TEETH AGAIN—REPEATED DRESSINGS.

BY J. W. WASSALL.

We have dead dentine infiltrated with matter highly irritating and poisonous to living tissue in intimate contact with vital cementum, which, in turn, is closely enveloped in pericementum.

What must the effect be on the cementum and peridental membrane of the materias morbi which are present in the underlying dentine?

There is no escape from the conclusion that it must account for many morbid conditions and symptoms, the etiology of which is otherwise obscure. There are, no doubt, exceptions of teeth in this state which cause no discomfort. But a little more time may prove that even these cases will not continue quiescent.

Unquestionably, this condition is responsible for numerous affections, more or less difficult of diagnosis, varying from necrosis to remotely situated abscesses, the only subjective symptoms in the causative tooth, discoverable, being a slight sense of lameness in mastication or to palpation.

What treatment does this pathological state indicate? It will not be uninteresting to first notice some of the methods which have, of late, been prominently set forth: The various mummification processes are only temporarily effective because, to use Dr. Harlan's words, "they will not stay mummified."

Emil Schrier destroys pulp-remains with a mixture of sodium and potassium, which is objectionable on the ground that the action of the drug does not extend into the entire depth of the tubules.

Professor Frank Abbott has written a work on "Dental Pathology and Practice" which will be widely read. It is peculiarly unfortunate for the younger members of the profession, whose methods are most likely to be influenced by it, that the chapter devoted to the consideration of this question, should be so inconsistent with modern bacteriological knowledge. Dr. Abbott imparts a lucid understanding of the fact of putrid dentine and the conditions to be overcome, but the treatment proposed is contrary to the commonest laws of asepsis. The triumph of modern surgery is secured by striving to prevent the entrance of germs into, rather than their destruction after admission to, a wound. How hazardous to teach that a pulpless tooth may be operated upon without first adjusting the rubber dam. The neglect of this precaution against the ingress of the myriads of mico-organisms, which are always present in the human mouth, is hardly excusable at this day. Dr. Abbott also recommends the use of bichlorid solutions for syringing out the pulp-debris. "This is of doubtful utility, because it is at once precipitated in the presence of albumen, thus losing its germicidal and antiseptic powers." (McFarland).

It is also a bad practice to pump zinc chlorid through a tooth to cauterize a pus-sac until a course of treatment for sterilization of the dentine with diffusible disinfectants has first been completed.

These few criticisms are submitted in order to substantiate the charge made at the opening of this paper, that much of the modern practice in the management of pulpless teeth does not conform to the present status of bacteriological science.

The successful treatment of teeth contaminated with putrid pulp-matter would to my mind, seem to depend upon the strict observance of two details of procedure: First, the exclusive use of diffusible disinfectants.

Second, the repeated and continued application of the disinfectant dressings for a sufficient length of time.

The reason why I give diffusible disinfectants the preference over coagulating drugs is because I am satisfied that exhibition at the pulpal orifices of dentinal tubules forms a plug of coagulated matter which prevents the further ingress of the disinfecting agent, imprisoning within the tubules putrefactive matter which will be a permanent source of irritation to the cementum and pericementum. If it is true, as maintained by Drs. Truman and Kirk, that the entire contents of the tubules is coagulated, there is nothing gained, for the resulting mass is suitable pabulum for micro-organisms, hence there would be no assurance that putrefaction would not recur within the tubules. In the investigations thus far published, the preponderance of evidence seems to be in favor of the avoidance of the coagulating drugs in the roots of pulpless teeth.

The second requisite to success in the disinfection of putrid dentine mentioned, was the element of time: How long shall a dressing be kept in a canal before it is proper to fill it? Until the dentine is permeated throughout its entire depth, and until all micro-organisms and their spores may reasonably be expected to be destroyed.

My observation are that these results are obtained in not less than twelve days—oftener sixteen or twenty—and in some few cases a little longer course of treatment is required. The dressings should be changed every four days until no stain or odor is perceived on the cotton-dressing other than the drug employed. Even though the dressing may come away clean on the fourth or eighth day, and you feel certain that the bacteria are killed, it is imperative that the use of the drugs be continued for the full period, in order to kill any spores which may be present, for they offer greater resistance to germicides, and hence require more time for their destruction.—*Dental Register*.

